# Inhibition and working memory capacity modulate the mental space-time association

**DOI:** 10.3758/s13423-024-02497-1

**Published:** 2024-04-19

**Authors:** Isabel Carmona, Jose Rodriguez-Rodriguez, Dolores Alvarez, Carmen Noguera

**Affiliations:** Department of Psychology, Health Research Center, University of Almería, Carretera Sacramento s/n. 04120 La Cañada de San Urbano, Almería, Spain

**Keywords:** Implicit processing, Mental timeline, Working memory, Inhibitory control, Individual differences

## Abstract

This research aimed to investigate whether the mental space-time association of temporal concepts could be modulated by the availability of cognitive resources (in terms of working memory and inhibitory control capacities) and to explore whether access to this association could be an automatic process. To achieve this, two experiments were carried out. In Experiment 1, participants had to classify words with future and past meanings. The working memory load (high vs. low) was manipulated and the participants were grouped into quartiles according to their visuospatial working memory capacity (WMC). Temporal concepts were displayed subliminally (immediate masking) and supraliminally (delayed masking). The ANOVA showed a performance pattern consistent with the left-past right-future conceptual scheme, regardless of both the type of masking and the working memory load, except in high WMC participants, in which, interestingly, the space-time association effect was absent. In Experiment 2, participants were asked to respond to the colour of the font of the temporal words, and their attentional control capacity was assessed. The results indicated a timeline effect that was irrespective of the WM load and the type of perceptual processing, but not of the WM capacity or the inhibitory abilities. These findings partially endorse the automatic and implicit access to the mental space-time association and suggest the involvement of the availability of cognitive resources. Individual WMC differences appear to modulate the automatic nature of the effect rather than the processing conditions themselves.

**Supplementary information:**

The online version contains supplementary material available at 10.3758/s13423-024-02497-1.

## Introduction

The mental representation of time has been the subject of research over the last decades, with the unanimous conclusion of the existence of an association between the mental representation of space and time. Indeed, the internal representation of time may be spatial in nature, as several lines of evidence suggest (Bonato et al., 2012). The SNARC (spatial–numerical association of response codes; Dehaene et al., 1993) and SPoARC (spatial–positional association of response codes; van Dijck & Fias, 2011) effects are two examples of how the cognitive system adds a spatial organization to presented information (Guida & Campitelli, 2019). In the first scenario, smaller magnitudes tend to elicit a quicker response from the left hand, whereas larger magnitudes lead to a faster response from the right hand. In the second case, the association is made between the left hand and the numbers presented in the first positions of a sequence shown in the presentation phase, and between the right hand and the numbers displayed in the last positions.

In this work, we focus on time-space interactions related to the tendency in Western cultures to associate the past with the left space and the future with the opposite space, and this association shall be referred as the mental timeline (MTL; Bonato et al., 2012). The space-time interaction is observed in both the processing of time-related concepts (e.g., yesterday–tomorrow; Santiago et al., 2011) and temporal durations (e.g., shorter–longer; Üstün et al., 2017). The theory of magnitude (ATOM) was described by Walsh (2003) and later reformulated by Bueti and Walsh (2009), as an alternative (or rather complementary) hypothesis to the MTL. This theory postulates a common neural system (brain regions such as the parietal and prefrontal cortex and cognitive mechanisms) for the processing of magnitudes concerning space, time, number and other dimensions, that embodies versatile or multi-duty neurons in the magnitude scaling system, allowing for the integration of spatial, temporal or size information through action. Neuropsychological evidence suggested some commonality in the location of lesions causing deficits in these dimensions. For example, several studies report that regions of damage that result in temporal deficits often also lead to deficits in spatial, numerical, and velocity perception (Cipolotti et al., 1991; Danckert et al., 2007; see also Bonato et al., 2016), suggesting overlapping mechanisms.

According to most studies on the topic, it seems evident that time concepts follow an ordered distribution, for instance, ranging from the oldest to the newest, from what has already happened to what is about to happen. The direction of writing in each culture probably mediates this distribution (He et al., 2021; Santiago et al., 2007).

The cognitive process underlying the serial order followed by different concepts (e.g., numbers or letters) has been described by Abrahamse et al. (2014) as serial order working memory (WM), by using the mental whiteboard metaphor, referring to the internal order in which concepts would be spatially coded. Based on the aforementioned work, the spatial attention system would play a fundamental role in serial order WM, such that the stimulus in the context of a task could activate the internal order representation, triggering a top-down attentional processing, a stimulus-driven or endogenous process (Capizzi et al., 2023; Corbetta & Shulman, 2002). The involvement of spatial attention on the representation of subjective time has been proposed by Weger and Pratt (2008), and they suggest that spatial attention could modulate performance in detection tasks requiring single-handed responses (e.g., the past meaning of concepts could direct attention towards the left-hand side, while the future meaning could lead to the right-hand direction). The key role of spatial attentional mechanisms in the processing of temporal concepts is supported by data from patients with neglect, a disorder of spatial awareness that affects contralesional spatial processing and representation (Bonato et al., 2016; Saj et al., 2014), especially in accessing numerical magnitude upon the mental number line (Umiltà et al., 2009; Zorzi et al., 2012). Bonato et al. (2016) mentioned that some authors even suggest WM and its interaction with spatial attention are one of the main determinants of these effects (Doricchi et al., 2005; van Dijck et al., 2012).

The literature provides the theoretical assumption of automatic activation of the mental spatial-temporal schema mediated, among other factors, by writing direction if the context of the task does not force other spatial coding (Bonato et al., 2012; see also Casasanto & Bottini, 2014). Based on Guida and Campitelli’s (2019) parsimony principles, it is proposed that the cognitive system relies on pre-existing schemas in long-term memory, such as an individual’s culture-specific reading and writing orientation, to spatialize the information.

Moreover, Shiffrin and Schneider (1984) provided a broad definition of automatic processes, suggesting the existence of unconscious and conscious processing. In the former, information is automatically activated, while the latter is closely related to strategic processes that depend on volitional control (Posner & Snyder, 1975).To the extent that an automatic process demands fewer cognitive resources (Ortells et al., 2006; Shiffrin & Schneider, 1984), it is worth asking whether activating and accessing this spatial-temporal representation would consume cognitive control resources, and, if so, what would happen when these resources are more limited (or less available), such as when WMC is low or there is a high mental load. Several authors have reported that measuring distractor interference under varying working memory load (e.g., high vs. low), can lead to worst responses when a to-be-ignored distractor is present under high memory load. In contrast, attentional resources can be distributed to ignore distracting information (e.g., through the action of inhibitory mechanisms) under low mental load (de Fockert, 2013; Ortells et al., 2016).

On the other hand, some researchers have shown that the individual differences in WMC can modulate performance in selective attention tasks (Conway et al., 1999; Ortells et al., 2016; Megías et al., 2020). In the study by Ortells et al. (2016), for example, participants with high versus low WMC were instructed to either attend to or ignore a briefly shown prime word, followed by either a semantically related or unrelated target on which they made a lexical decision. The ignored primes gave rise to reliable semantic negative priming only for high-capacity participants and an opposite effect (positive priming) for low-capacity participants. WMC is typically measured with complex span tasks, such as the Automated Operation Span task (AOSPAN; Unsworth et al., 2005) or the Automated Symmetry Span task (ASYMSPAN; Unsworth et al., 2009), as well as simple span tasks such as the Digit Backward Span (DBS; Engle, 2002; Hilbert et al., 2015). Some authors suggest that the Visual Change Location Task (Johnson et al., 2013) is the most appropriate task to assess visual-spatial WMC. Empirical evidence indicates that the storage capacity measures obtained from this task provide a useful index of visuospatial WMC (Johnson et al., 2013; Vogel & Machizawa, 2004), which is related to broader measurements of cognitive capacities (Fukuda et al., 2010).

### Current study

The aim of this study was twofold: firstly, to explore, for the first time, how the availability of cognitive resources (i.e., visuospatial WM and inhibitory control) affects the activation of the mental space-time association, and secondly, to determine whether access to the mental timeline could be an automatic process. To achieve this, two experiments were designed. In Experiment 1, participants responded to whether a word in the centre of the screen referred to the past or the future by pressing the appropriate response key (G, for left hand; L, for right hand). The working memory load was manipulated in this experiment along with two masking conditions, a mask presented immediately after the word (subliminal perception) or after a delay (supraliminal perception). Furthermore, the visuospatial WMC was evaluated using the Visual Change Location Task (Johnson et al., 2013). A pattern of data consistent with the left-past right-future conceptual metaphor is expected to be observed in both subliminal and supraliminal processing conditions, regardless of the WM load, since an automatic process requires fewer cognitive resources (Ortells et al., 2006). To the extent that the mental scheme of time concepts (e.g., left-past right-future) is spontaneously activated, individual differences in WMC could modulate the timeline effect, particularly when participants are required to respond with an opposite schema (left-future right-past) and, therefore, need to ignore or inhibit the more automatic schema.

In Experiment 2, the participants completed a conflict task in which they responded to the font colour (e.g., blue) of temporal words, regardless of their meaning (past, future or present). The inhibitory control capacity (Stroop interference) of the participants was evaluated. As previously stated, it was hypothesized that the inhibitory capacities could affect the timeline pattern, such that the participants with a high Stroop interference score would show a high mental timeline effect (Experiment 2). This task includes words related to the present time to explore whether subjective time is spatially represented on a line, where time is perceived as running from one end to the other, as suggested by previous research (Bonato et al., 2012; Santiago et al., 2007).

If these hypotheses are fulfilled, the results would support the idea that the mental timeline, despite being an automatic effect, depends on the availability of cognitive resources, since cognitive control mechanisms would be essential to prevent this activated automatic representation (left-past right-future) from interfering with the activation of another schema (left-future right-past; Experiment 1), or task switching (naming the colour rather than the meaning; Experiment 2).

## Method

### Participants

A sample of 187 healthy undergraduates (from the University of Almería) participated in the study: 141 in Experiment 1 (M = 23.9; SD = 8.4), and 46 in Experiment 2 (M = 22,1; SD = 5.1). Participants in Experiment 2 were different from those in Experiment 1. All participants had normal or corrected-to-normal vision. The volunteers signed an informed consent protocol in accordance with the Declaration of Helsinki for biomedical research involving humans and the university’s bioethics regulations (UALBIO2022/050). The collected data were handled correctly in compliance with the Organic Law 3/2018 of the Spanish State, dated 5 December 2018, on the Protection of Personal Data and Digital Rights Guarantee Act.

Sensitivity analyses were performed with the G*Power software 3.1.9.2 (Faul et al., 2007) to determine the minimum effect size that could be reliably detected from the sample size in each experiment, with α = .05, and power of .99. The minimum effect size was d = .24 for Experiment 1 (with a sample size of 141), and d = .31 for Experiment 2 (with a sample size of 46). Post hoc power analyses from our data showed that the minimum effect size was .47 and .59 for main effects (Experiments 1 and 2, respectively), and .25 and .33 for interactions (Experiment 1s and 2, respectively).

### Materials

The experiments were designed using E-Prime v3.0 (Psychology Software Tools, Pittsburg, PA, USA) software, which also enabled the recording of data. The lighting of the site was controlled and a computer (6000 MB of RAM) with a 17-in. monitor (640 × 480; 60 Hz) was always used to run the experiments. The QWERTY keyboard was used in all experiments.

For Experiment 1, a total of 40 words were selected from a previous study by Santiago et al. (2007), 20 with past meaning (e.g., yesterday) and the rest with future meaning (e.g., tomorrow). A white cross (+) centred on the screen on a black background in Courier New font size 18 was displayed as a fixation point. The words with past or future meaning were displayed in capital letters in the same font and in white; the letter mask was made up of random uppercase consonants (e.g., DMNTSRPL), varying in length from four to eight letters, depending on the length of the word to be masked. In Experiment 2, a set of 30 words were selected from Experiment 1 (15 with past meaning, and 15 with future meaning), adding another 15 words with present meaning (e.g., today). These stimuli were presented in lower case, size 18 in Courier New font, on a black background in either blue or red. The full list of stimuli used in both experiments is available as Online Supplementary Material (OSM).

### Procedure

At the beginning of the experiments, the instructions were explained orally to the participants and displayed on the screen.

#### Experiment 1

Participants were instructed to decide whether the masked words were related to the future or to the past, and to respond as quickly and accurately as possible. The participants used their two index fingers on the G and L keys to indicate whether the words referred to the ‘past’ or ‘future’. The response-key meaning (G for past or future; L for past or future) differed across the two task blocks, and the order of the blocks was counterbalanced across participants.

**Fig. 1:**
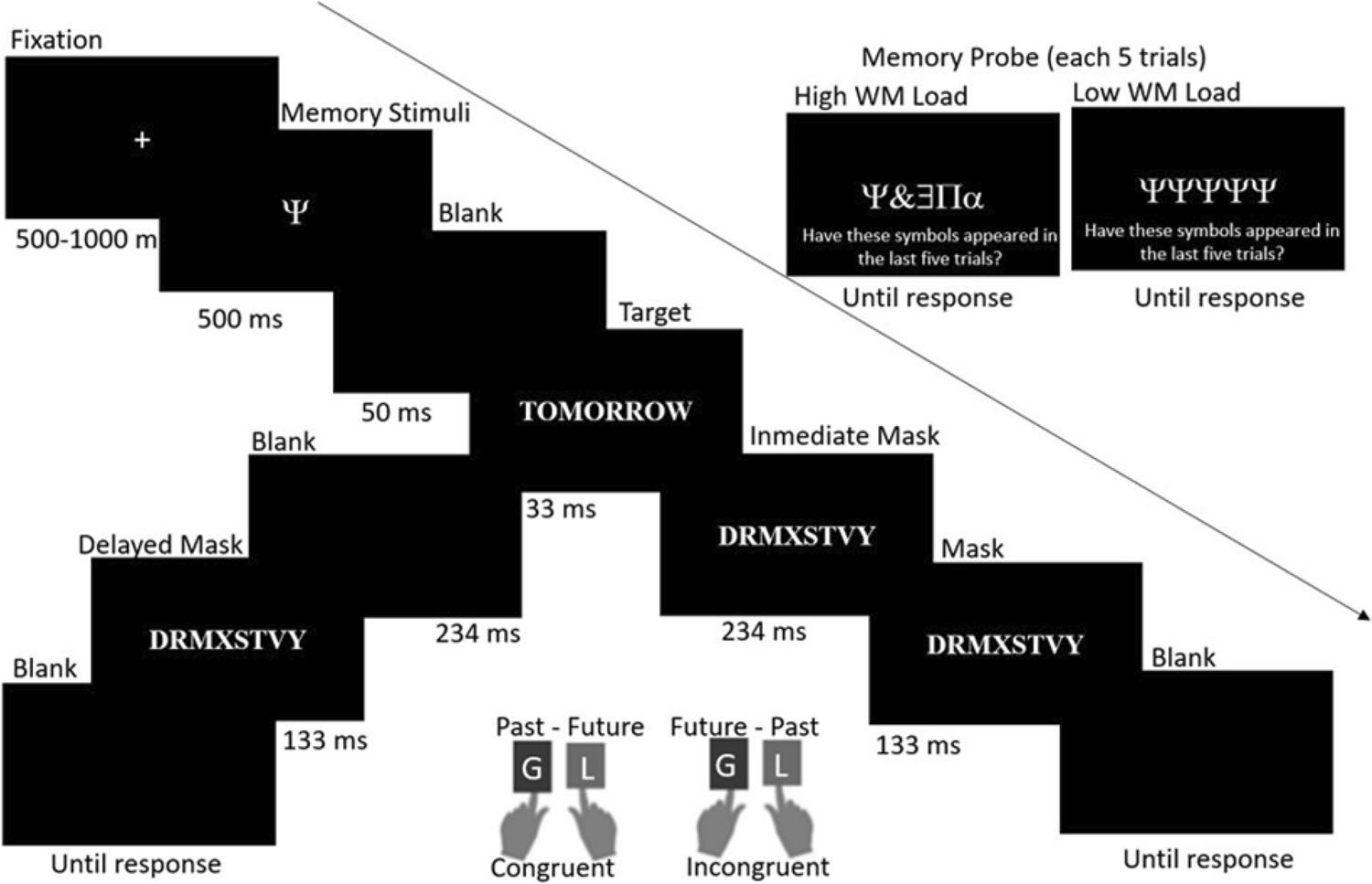
Temporal sequence of event of a trial (immediate and delayed masking conditions), congruent response hand (left-past, right-future) and incongruent response hand (left-future, right-past). The target could be preceded by the same memory stimuli in five trials (low load), or by a different symbol in each of the five trials (high load). Memory probes were displayed after every five trials

The sequence of events in one trial can be seen in Fig. 1. The fixation point (+) was randomly presented for 500 ms or 1,000 ms in the centre of the screen, followed by a symbol to be memorised for 500 ms (e.g., $), randomly selected from six different symbols ($@ &%#∏) in every trial under the high WM load condition. In the low WM condition, the symbol was the same in all trials. After a 50-ms blank screen, the target appeared for 33 ms, either immediately followed by a letter mask for 367 ms (immediate masking condition), or by a blank screen for 234 ms and letter mask for 133 ms (delayed masking condition). Thus, the interval between the onset of the target and the onset of the response display (stimulus onset asynchrony, SOA) was 400 ms in both masking conditions. The letter mask appeared at the same location as the target. Finally, a blank screen was presented until response, or up to 5,000 ms, whichever came first. After five trials, a screen was displayed that contained five symbols, which could be identical (e.g., ‘$$$$’; low WM load condition) or different (e.g., ‘$ @ $%#’; high WM load condition). Participants were asked to decide whether the symbols had previously appeared in the same order by pressing either the 1 key to respond ‘yes’, or the 2 key to respond ‘no’.

The participants first completed an eight-trial practice block and then two 160-trial experimental blocks, which differed in key meaning-response assignments (G for past or future; L for past or future). The order of the blocks was counterbalanced among the participants. In each block of trials, the mask appeared immediately in 80 trials, generating a condition where the target was unnoticed by the participants (immediate mask condition). In the other 80 trials, the target remained clearly visible (delayed mask condition). The words with a past meaning appeared in 40 of the trials, and those with a future meaning in the remaining trials. The congruency of these conditions was defined by its consistency with the left-past right-future conceptual metaphor: Response-hand congruent with the mental scheme (left-hand response to past, right-hand response to future).

At the beginning of the experiment, participants performed a recognition task to determine the objective visibility threshold of the displayed stimuli. The target words, presented in the centre of the screen, were categorised by the participants as nouns or verbs. They responded by pressing either the '6' or '7' keys on the standard keyboard using the index and middle fingers of the same dominant hand. The task consisted of 40 trials in total, with 20 trials assigned to each masking condition. Ten words were past tense (five verbs and five nouns), while another ten were future tense, half verbs and half nouns. The masking condition was the same as in the experimental blocks.

##### Change Location Task

WMC was assessed using the Change Localization Task (Johnson et al., 2013). A white fixation point appeared in the centre of a black background screen for 1,000 ms. Then four coloured circles (blue, green, orange, red, green, yellow, cyan, magenta, white and black) were shown for 150 ms, at an angular distance of 3.36 ° and 6.24° from the fixation, without repetition in the same display. After a 900-ms blank screen, a set of four circles appeared positioned identically, three in the same colour. The participants were required to select the changed colour by clicking on it with the mouse. The total number of trials was 64 (for more details, refer to Castillo et al., 2021; Noguera et al., 2019). Mean correct response scores were transformed to a K-index based on the Pashler-Cowan equation (see Cowan et al., 2005).

#### Experiment 2

##### Time-colour task

The participants were instructed to indicate whether the display words (with past, future or present meaning) were coloured red or blue, as quickly and accurately as possible. They used their index fingers to press the G or L keys to identify the colour of the words as ‘red’ or ‘blue’. The response-key assignment was counterbalanced between participants. The task consisted of a single block of 180 trials (15 words with past meaning, 15 with future meaning, and 15 with present meaning, repeated twice per colour). The words were randomly displayed, with half of the trials coloured blue and the other half red. The congruence of the trials was defined as follows: for congruent trials, the word with past meaning was presented in a colour, the response key for which (response hand) was on the left side (G: red or blue), while the future and present meanings were displayed in a colour corresponding to the response hand on the right side (L: red or blue); for incongruent trials, the response hand for the future and present meanings was on the left side, while the response hand for the past meaning was on the right side.

**Fig. 2:**
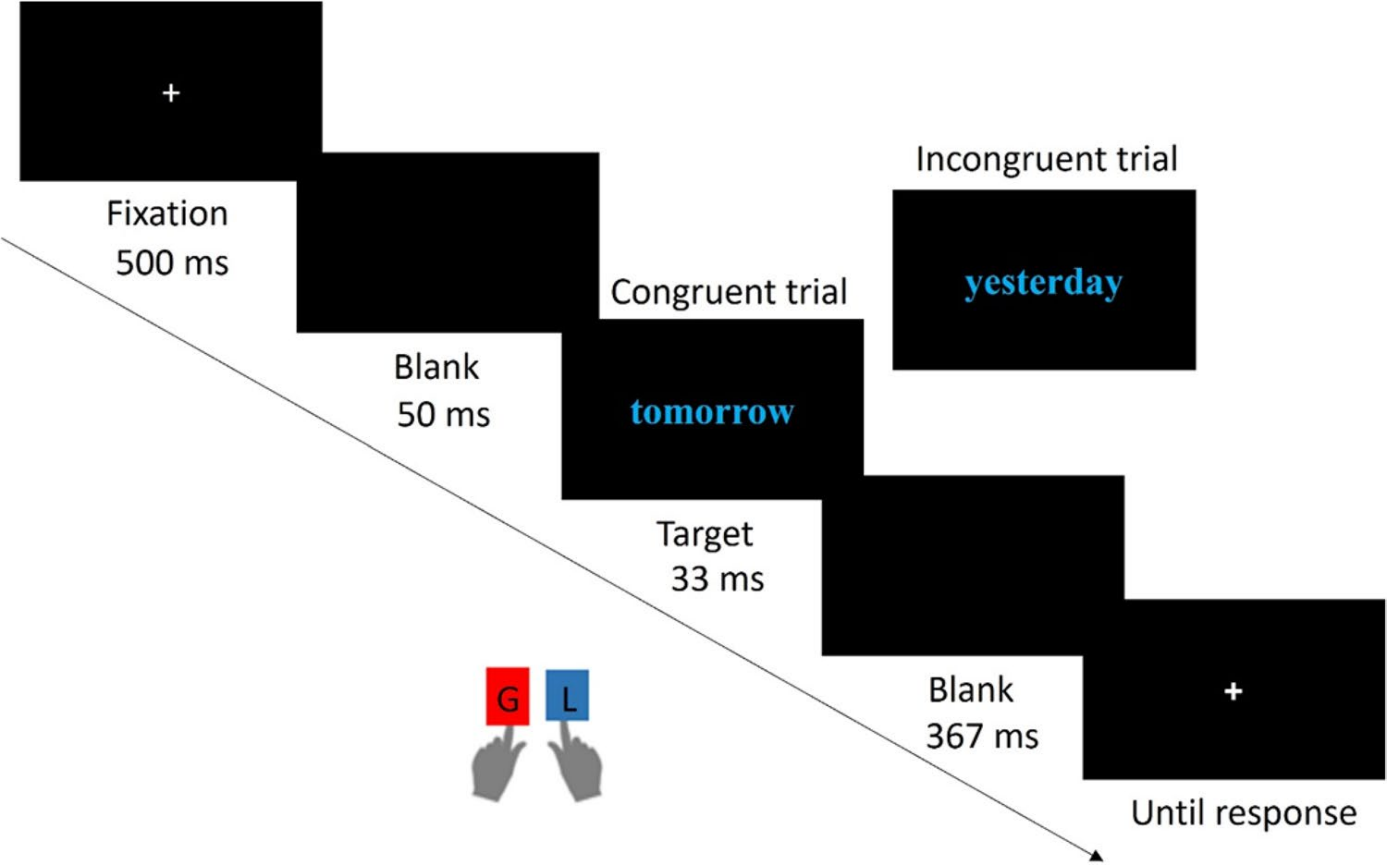
Temporal sequence of events of a trial of the Time-Colour Task in Experiment 2

Each trial began with a central cross as a fixation point, which lasted between 500 and 1,000 ms. A word representing past, present or future time (e.g., ‘yesterday’, ‘today’ or ‘tomorrow’) in either red or blue was presented on the screen for 33 ms before a 367-ms blank interval. Subsequently, the response screen was presented until a response was submitted. The participants were instructed to identify the colour of the font in which the word was written. Finally, a blank screen was presented for 500 ms (see Fig. 2).

##### Stroop task

In addition to the task described above, Experiment 2 included a computerised version of the Stroop task. Each trial began with a central cross on the screen with a duration varying from 600 to 1,000 ms. Then, a colour word (red, green or blue) appeared in the centre of the screen. Participants were required to recognise the font colour in which the word was printed. The task consisted of three practice trials followed by a block of 60 trials. Of the trials, 70% were congruent (i.e., meaning-colour matched; RED printed in red), and the remaining 30% were incongruent (meaning-colour mismatched; RED printed in blue).

### Data analysis

#### Experiment 1

Reaction times (RTs) for correct responses were recorded and analysed as a function of five factors in a repeated-measures analysis of variance (ANOVA): Response mapping (congruent or incongruent) × Tense (past or future) × Masking (immediate or delayed) × WM Load (high or low) and WM Capacity group (quartiles Q1, Q2, Q3 and Q4). Lineal regression analyses were conducted to examine whether WMC (K index) affects the mental timeline effect.

The discriminability index was calculated from the data of the recognition task in Experiment 1 for the two masking conditions, according to the equation d’= ZHits – ZFA (FA = false alarm; Di-Russo et al., 2016; MacMillan & Creelman, 1990). Chance-level discrimination was d’ = 0.

#### Experiment 2

RTs on correct responses were recorded and analysed using an ANOVA with two factors: Response mapping (congruent, past-future, or incongruent, future-past) × Tense (past, present, or future). Lineal regression analyses were performed to determine whether the inhibition capacity (Stroop interference), defined as the difference between RTs in incongruent trials and congruent trials, influences the mental timeline effect.

The Kolmogorov-Smirnov test and Levene’s test were conducted to check normality of data and homogeneity of variance, respectively. Results showed the normal distribution of data and the homogeneity of variance in all variables. Latencies larger than 2.5 standard deviations above the means were excluded from the analyses (2.5% in Experiment 1; and 1% in Experiment 2). An alpha of .05 was used for tests of statistical significance. Bonferroni correction was applied in multiple post hoc comparisons.

## Results

### Experiment 1 (working memory (WM) load and capacity)

The ANOVA (Response mapping × Masking × WM Load × Tense × WM Capacity group) from RT showed a main effect of Response mapping [*F* (1,134) = 29.3; *p* < .001; *ηp*^*2*^ = .18]; participants were faster to respond in the congruent trials (past-left and future-right; 778 ms, SD =22.6) than in the incongruent trials (past-right and future-left; 942 ms, SD = 31.6). The effect of WM Load was also significant [*F* (1,134) = 48; *p* < .001; *ηp*^*2*^ = .26]; RTs were lower in the high-load condition (829 ms, SD = 23.7) than in the low-load condition (892 ms, SD = 24.2). The main effect of Tense reached significance [*F* (1,134) = 6.7; *p* = .011; *ηp*^*2*^ = .06]; responses to past words were slower (876 ms, SD = 21.1) than to future words (844 ms, SD = 24,3). Furthermore, a main effect of Masking was found [*F* (1,134) = 5.7; *p* = .019; *ηp*^*2*^ = .043]; RTs were higher in the immediate masking condition (873 ms, SD = 23.8) than in the delayed masking condition (830 ms; SD = 24.8)

**Fig. 3:**
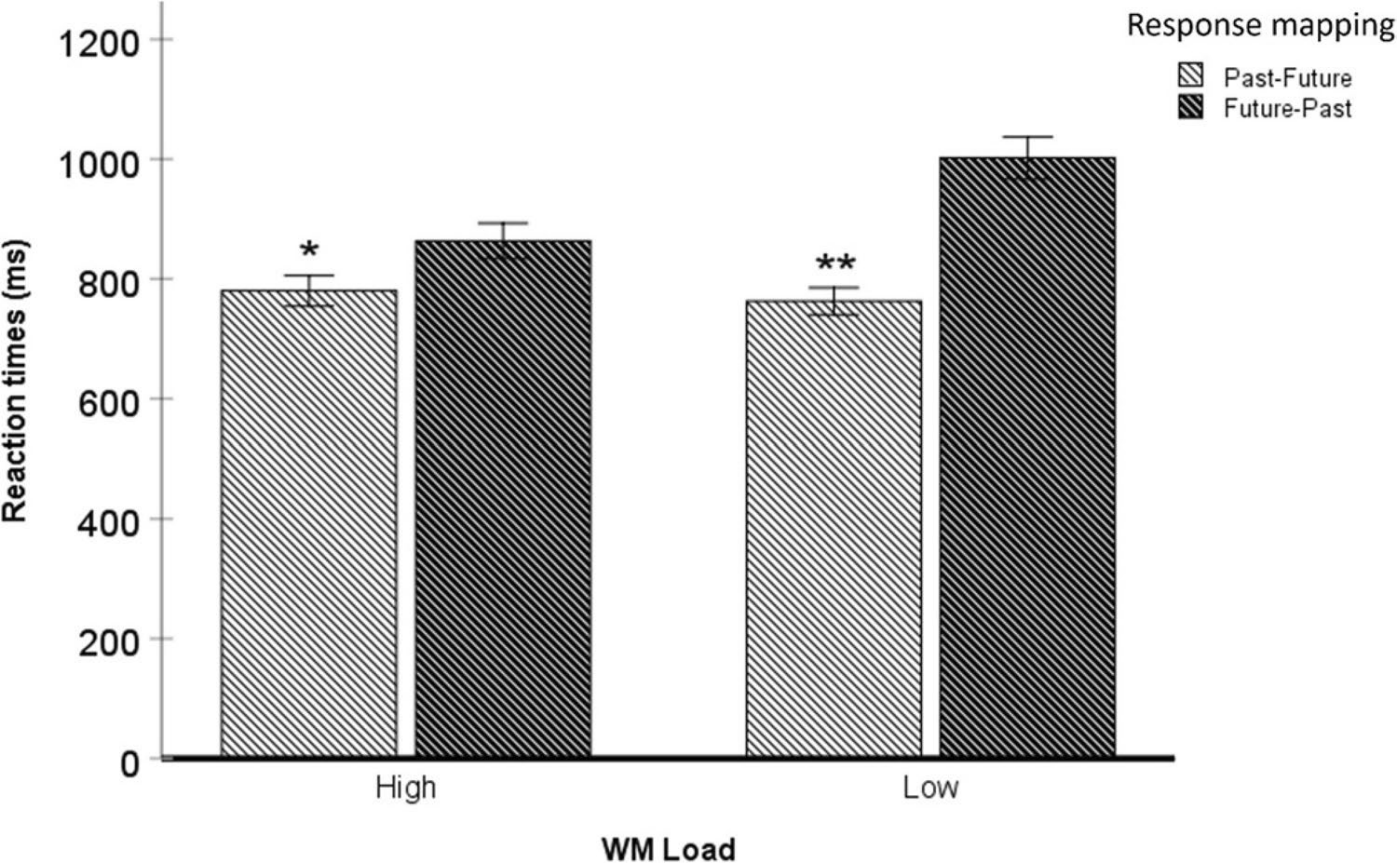
Mean reaction times (in ms) as a function of Working memory (WM) load (high, low) and Response mapping (Congruent, past-future; Incongruent, future-past) in Experiment 1. * p < .01; ** *p* < .001. Error bars represent standard deviation

fThe ANOVA also showed a two-way interaction between the WM Load and Response mapping [*F* (1,134) = 88.7; *p* < .001; *ηp*^*2*^ = .40], indicating the Response mapping effect with the temporal mental schema in both conditions of WM Load (see Fig. 3). Under high WM Load conditions, the participants responded faster in line congruent trials (780 ms, SD = 24.7) compared to incongruent trials (863 ms, SD = 23.4), [*F* (1,134) = 8.4; *p* = .004; *ηp*^*2*^ = .06]. A similar pattern was found in the low WM Load [ *F* (1,134) = 49.1; *p* < .001; *ηp*^*2*^ = .26], with faster RT to congruent line trials (763 ms, SD = 22.4) compared to incongruent trials (1,002 ms, SD = 32.1).

**Fig. 4:**
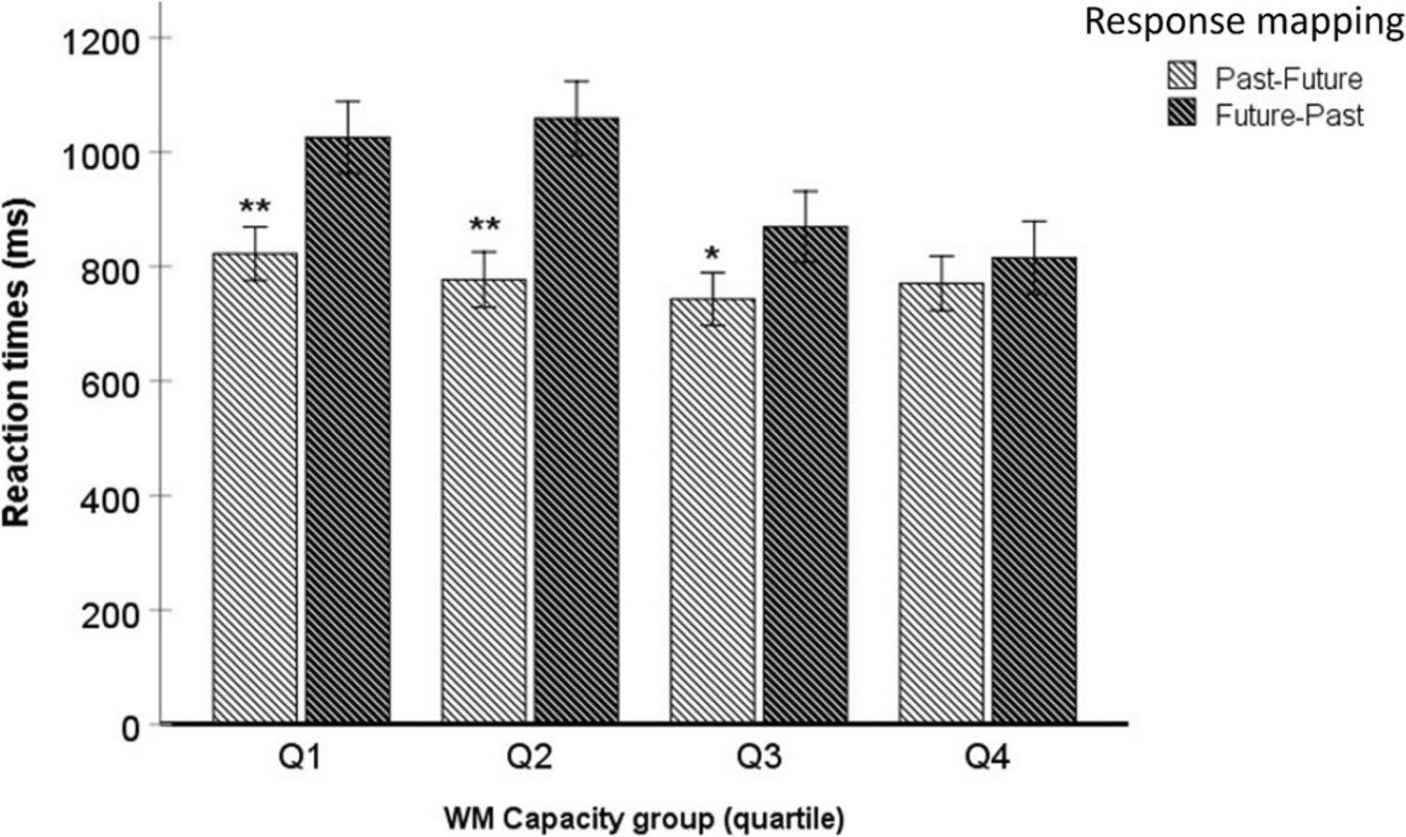
Mean reaction times (in ms) as a function of Response mapping (Congruent, left-past, future-right; and Incongruent, future-left, past-right) and Working memory capacity (WMC) group (quartiles Q1, Q2, Q3 and Q4)*.* * *p* < .01; ** *p* < .001. Error bars represent standard deviation

Finally, a two-way interaction was found between the WMC Group and the Response mapping [*F* (3,134) = 2.84; *p* = .04; *ηp*^*2*^ = .06]. A statistically significant effect of Response mapping was observed in WMC groups Q1 [*F* (1,34) = 12.3; *p* = .001; *ηp*^*2*^ = .27], Q2 [*F* (1,32) = 14.5; *p* = .001; *ηp*^*2*^ = .32] and Q3 [*F* (1,35) = 4,21; *p*=.048; *ηp*^*2*^ = .11]. In contrast, WMC group Q4 did not show significant differences between the response hand to congruent trials and to incongruent trials (*p* =.53). See Fig. 4 for more details. No others main effects nor interactions were found (*p*s > .05).

**Fig. 5:**
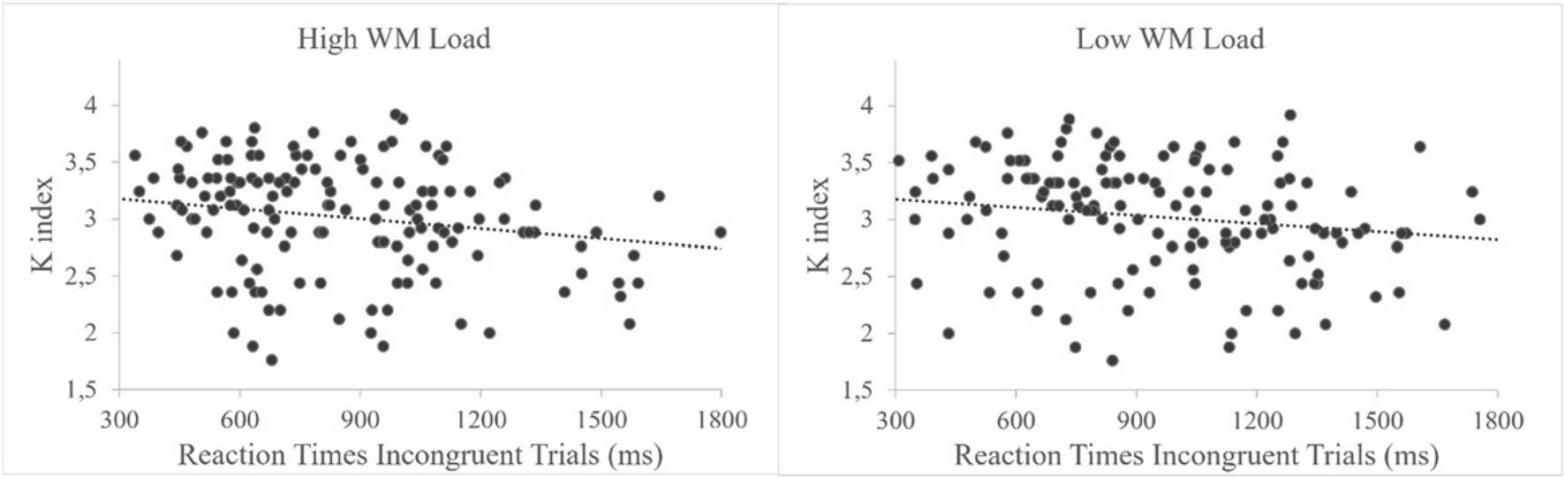
Mean reaction times (in ms) for incongruent trials (left-future, right-past) as a function of the working memory (WM) capacity (K index) and WM load condition (high and low)

Further regression analyses showed that the WMC (measured by K index) predicted the latencies for incongruent trials in both high WM Load condition [R-squared = .05, *p* = .01] and low WM Load condition [R-squared =.041, *p* = .015]. See Fig. 5.

### Recognition task

A high discriminability index was obtained in the delayed masking condition (d’ = .1.3), significantly above the chance level [*t* (134) = 8.5, *p* = .001], compared to the chance level achieved in the immediate masking condition (d’ = .08; [*t* (134) = .6, *p* > .05].

### Memory probe (accuracy)

A main effect of WM load [F(1, 141)=10.3, p= .002, ηp^2^ = .07] was found. Bayes factor, F01 = .11, showing moderate evidence against the null hypothesis. The percentage of correct responses was 75% in the High memory load, and 81% in the Low memory load. This result shows the effectiveness of the load.

### Experiment 2 (inhibition capacity and timeline effect)

**Fig. 6:**
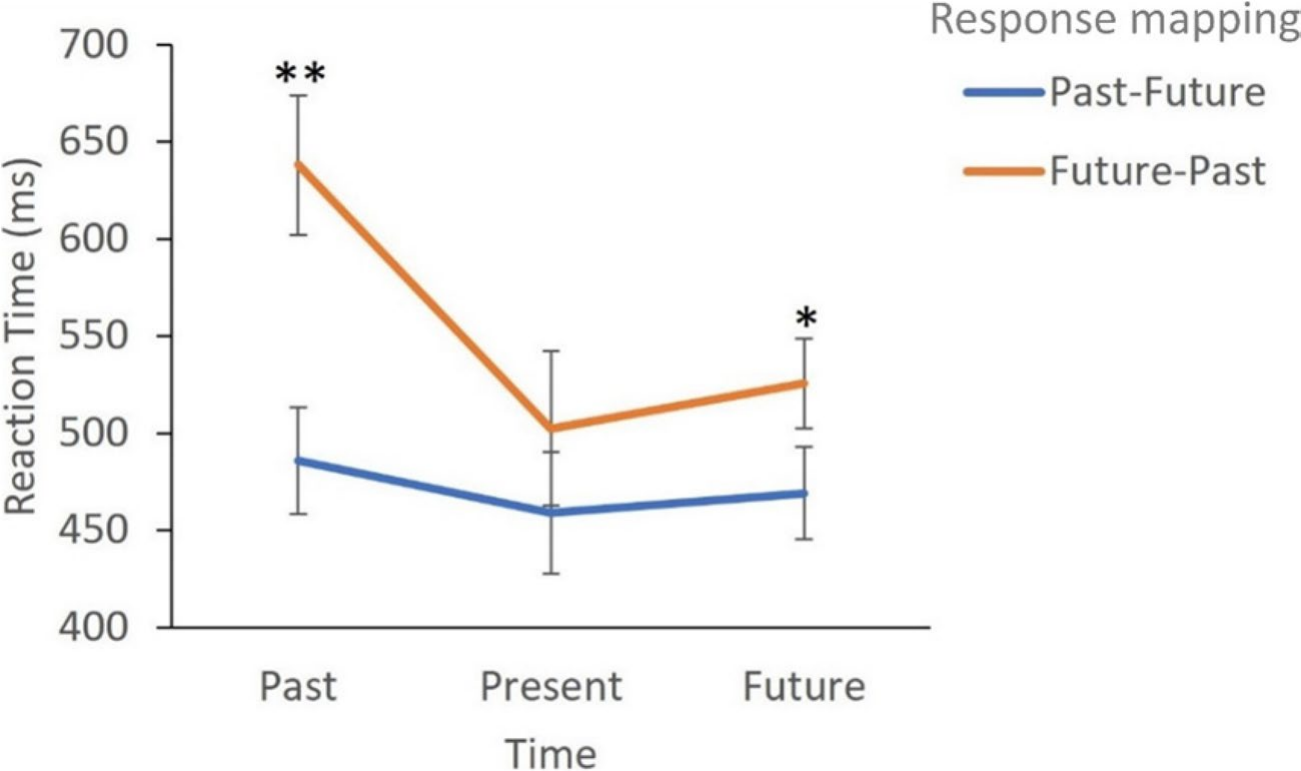
Mean reaction times as a function of Response mapping (Congruent, past-left, future-right, present-right; Incongruent, past-right, future-left, present-left). * *p* < .01; ** *p* < .001. Error bars represent standard deviation

The ANOVA Response mapping × Tense analysis on RTs showed a statistically significant main effect of Response mapping [*F* (1,44) = 15,4; *p* < .001; *ηp*^*2*^ = .26]; congruent responses (left-past, right-future) were faster (471 ms, SD = 22,5) than incongruent responses (555 ms, SD = 27,6). Also, a main effect of Tense was observed [*F* (2,88) = 4,3; *p* = .016; *ηp*^*2*^ = .10]. Overall, responses to past tense were significantly slower (562 ms, SD = 23) compared to present tense (481 ms, SD = 31, *p* = .020). No significant difference was found between the past and the future tense (562 ms vs. 498 ms, respectively, SD = 29, *p* = .13), nor in responses to the future or present tense (*p* = 1). Although no interaction effect was found (*p* > .05), additional analyses showed that the effect of Response mapping was statistically significant for responses to the past tense (*p* < .001) and the future tense (*p* = .004), but there was no significant effect for the present tense (*p* < .05), as can be seen in Fig. 6.

**Fig. 7:**
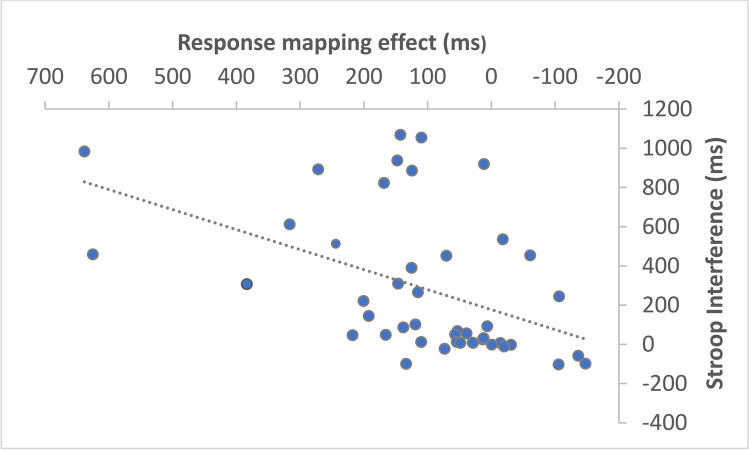
Response mapping effect (differences on response times between incongruent trials, future-past, and congruent trials, past-future) as a function of the Stroop interference

Further linear regression analyses showed that the inhibition capacity (Stroop effect) was a predictor of the Response mapping effect [R square = .21, *p* = .002]. Figure 7 shows the relationship between the Response mapping effect and the Stroop interference: a high Stroop interference score was found to be associated with a high Response mapping effect.

## Discussion

Temporal words were presented in the centre of the screen, and participants responded bimanually (on each answer key, the index finger of each hand; G, left hand; L, right hand) based on task instructions. Participants’ performance depended on the response mapping (congruent, past (G)-future (L), or incongruent, future (G)-past (L) and the tense of the words in in both experiments, matching the left-past, right-future internal schema typical of literate Western cultures (Chae & Hoegg, 2013; Santiago et al., 2007; Starr & Srinivasan, 2021).

To our knowledge, no prior studies have demonstrated the involvement of attentional mechanisms in the space-time association beyond proposing a theory (Weger & Pratt, 2008). According to Abrahamse et al. (2014), the mental whiteboard hypothesis on serial order in WM proposes that each element of a sequence is linked to a specific position marker that will be remembered later. In this context, i.e., when a verbal serial order (such as time words) is being processed, each element appears to be associated with particular internal spatial templates. These links would rely on past experiences, creating a specific connection between each item and a relevant spatial coordinate. This process of binding is carried out in working memory. It is probable that this framework (template) is activated automatically as soon as we observe a temporal sequence that can be arranged. Mental representation of time may involve visuospatial attention, since the mental schema (past left, future right) could be activated implicitly with a sequence of temporal concepts of trials; this mental whiteboard would trigger a kind of internal ‘Simon effect’, in which responding with the hand that coincides with the side of the mental ‘screen’ on which the stimulus appears would be facilitated (Hommel, 1993; Simon & Rudell, 1967; Simon, 1969). If the demands of the task conflict with the internal time-space schema, inhibitory control mechanisms could be activated to implement a new response schema.

Importantly, this pattern depends on the availability of cognitive resources. Several studies have reported that expectancy-based strategic processes are modulated by WM, as these processes consume some of the cognitive resources available in this memory system. The authors of these works found worse performance under conditions of high (vs. low) load (de Fockert, 2013; Ortells et al., 2017), and/or in people with low (vs. high) WMC (Fernández et al., 2021; Megías et al., 2020). If the activation of this mental timeline is automatic and thus requires minimal attentional resources, it should not be affected by the greater or lesser availability of these resources. The results of Experiment 1 were consistent with this hypothesis. In general, there was a consistent effect of the congruent response hand, regardless of the WM load: participants responded faster in the congruent left-past right-future condition compared to the left-future right-past condition, which was considered an incongruent schema. It should be noted that the high-load condition affected their performance, resulting in faster responses compared to the low-load condition. This may be due to a greater demand for cognitive resources. Faster responses under these conditions could be the result of a mechanism for discharging memory, facilitating the upgrade process in working memory to prepare for the next trial. The participants were required to hold five different symbols in their WM until the recognition display appeared, while simultaneously classifying the time words. The high demands on resources did not affect the activation and maintenance of the mental timeline to perform the task. However, inhibiting the prepotent schema in response to opposite or different task demands (left-future right-past) was not effective, thus resulting in the observation of the timeline effect once more (i.e., slower responses to the incongruent schema than to the congruent schema).

Our data in Experiment 1 revealed an interesting result: WMC modulated the effect of timeline in both WM load conditions. Specifically, the effect of space-time association was absent in participants with a higher WMC (quartile Q4). The findings of Experiment 2 indicate that attention control mechanisms influenced the space-time effect, with higher Stroop interference scores relating to a stronger effect. Furthermore, a notable result was the observation of the timeline effect when recognising the font colour of temporal words. This again confirms the existence of an automatic space-time association.

Different cognitive processes contribute to visuospatial memory performance, so the visual change location task (Fukuda et al., 2010) not only assesses the maintenance of item representations from the memory array, but also reflects the executive processes involved in performing the task, such as visuospatial attentional mechanisms (Vogel & Machizawa, 2004). Otherwise, the attentional control mechanisms underlying the Stroop effect are well known (Burgoyne et al., 2023). Taken together, these data support the automatic nature of this effect and the notion that cognitive resource availability plays a crucial role in this effect.

Moreover, the study of the spatial conceptual representation of time has usually been approached under conditions in which stimuli are consciously perceived. The masking paradigm used in this study, adapted from that of Ortells et al. (2006, 2012), also allowed for the presentation of the words under conditions of non-conscious (subliminal) and conscious (supraliminal) perception. The participants responded congruently with the left-past right-future mental schema in both conscious and nonconscious processing conditions. Recognition task discriminability index values confirm that delayed masking (conscious perception with discriminability index above chance level) and immediate masking (non-conscious perception with discriminability index near chance level) have different discriminability. This finding provides clear evidence supporting the implicit activation of this schema, as the subliminal presentation of the words prevents the conscious use of a strategic or expectancy-based response.

In conclusion, the current data indicate the automatic nature of the left-past and right-future mental scheme. This association is observed irrespective of the processing condition. Our study provides further evidence in support of the implicit mechanism of the mental timeline. Most importantly, our data suggest the involvement of attentional mechanisms in this space-time association.

This procedure could be a useful tool for exploring whether this internal representation remains stable over an individual’s lifespan or whether it is affected in individuals with laterality or reading problems (e.g., dyslexia). In this context, it is hypothesised that the above populations, as well as those with spatial and/or temporal pathologies that may alter this mental schema, may benefit indirectly from restoring or rehabilitating the temporal mental line. To address these issues, further research is required.

## Supplementary information

Below is the link to the electronic supplementary material.

Supplementary file1 (PDF 214 KB)

## Declarations

### Open practices statement and data availability statement

The data and materials for all experiments are available via the Open Science Framework at https://osf.io/cb8h4/.
