# Supplementary material for: Inhibition and working memory capacity modulate the mental space-time association
Source: Psychon Bull Rev. 2024 Apr 19;31(6):2634–45. doi: 10.3758/s13423-024-02497-1 (PMC11680673; doi:10.3758/s13423-024-02497-1)
Supplement: Supplementary file 1 — Supplementary file1 (PDF 214 KB) [file 13423_2024_2497_MOESM1_ESM.pdf]

Experiment 1. Bayesian factor analysis

Although the ANOVA results lacked reliable interactions between Response mapping, X Masking, and WM Load, we conducted Bayesian analysis to examine the Response mapping effect in each processing type and WM load condition, as suggested by the reviewer. The new analyses validate our previous findings, as reliable effects of Response mapping were identified in the low WM load condition, in both the delayed and immediate masking conditions, with robust evidence against the null hypothesis (evidence categories for the Bayes Factor (BF ij) as given by Jeffreys, 1961). Similar findings were observed in the high working memory load condition during delayed masking. A summary of these results is presented in the next table, which could be included as supplementary material.

Table 1.

RESPONSE MAPPING (congruent vs incongruent)	N	Mean diff.	Evidence	Bayes Factor (01)	t	gl	Sig. (bilateral)
DELAYED MASKING- HIGH WM load.	144	-110,4	Strong against null hyp.	,036	-3,559	143	,001
IMMEDIATE MASKING- HIGH WM load.	145	-54,5	Anecdotic against null hyp	3,846	-1,671	144	,097
DELAYED MASKING- LOW WM load.	145	-255,9	Strong against null hyp	,000	-6,413	144	,000
IMMEDIATEMASKING- HIGH WM load.	145	-231,5	Strong against null hyp	,000	-6,746	144	,000

Bayes factor: null hypothesis versus alternative.

Evidence Categories for the Bayes Factor (BF ij) as given by Jeffreys, 1961

Post hoc comparisons for critical interactions including, for Experiment 1, the comparison across the different WM capacities of the compatible/incompatible mappings.

Q4_Higher WM Capacity. Congruent vs. Incongruent Response mapping.

Bayes factor, F01= 6.331. Moderate evidence in favour the null hypothesis**.** $T(36)=.64$, $p=.53$

Q3_WM Capacity Bayes factor, F01= .835. Anecdotic evidence against the null hypothesis. $T(36)=2.2$, $p=.033$

Q2_WM Capacity
Bayes factor, F01= .013. Strong evidence against the null hypothesis. *T*(33)=4, *p*<.001

Q1_Lower WM Capacity
Bayes factor, F01= .021. Strong evidence against the null hypothesis**.** *T*(36)=4, *p*=.001

In addition, we have compared the performance between WM Capacity groups, in both Response mapping conditions, congruent and incongruent. The results show significant differences between higher WM Capacity group (Q4) and lower WM Capacity groups (Q1 and Q2) in the incongruent Response mapping condition. By contrast, there were no differences between groups in the congruent Response mapping condition, as can be seen in the next Tables.

Response mapping condition. Congruent

(I) CUARTIL_ WMC	(J) CUARTIL_ WMC	Mean diff. (I-J)	Error D.	Sig.
1,00	2,00	51,1651	66,36856	1,000
	3,00	93,7964	64,52255	,890
	4,00	46,8270	65,40077	1,000
2,00	1,00	-51,1651	66,36856	1,000
	3,00	42,6313	65,94905	1,000
	4,00	-4,3380	66,80852	1,000
3,00	1,00	-93,7964	64,52255	,890
	2,00	-42,6313	65,94905	1,000
	4,00	-46,9693	64,97501	1,000
4,00	1,00	-46,8270	65,40077	1,000
	2,00	4,3380	66,80852	1,000
	3,00	46,9693	64,97501	1,000

Response mapping condition. Incongruent

(I) CUARTIL_ WMC	(J) CUARTIL_ WMC	Mean diff. (I-J)	Error D.	Sig.
1,00	2,00	-23,0833	88,03777	1,000
	3,00	178,5113	85,58904	,233
	4,00	230,2606	86,75400	,049
2,00	1,00	23,0833	88,03777	1,000
	3,00	201,5946	87,48129	,136
	4,00	253,3439*	88,62138	,029

3,00	1,00	-178,5113	85,58904	,233
	2,00	-201,5946	87,48129	,136
	4,00	51,7493	86,18923	1,000
4,00	1,00	-230,2606	86,75400	,049
	2,00	-253,3439*	88,62138	,029
	3,00	-51,7493	86,18923	1,000
